# Cardio-Renal-Metabolic Syndrome with Emphasis on Chronic Kidney Disease: Educational Attainment and Progression—A Retrospective Cohort Study

**DOI:** 10.3390/healthcare13212671

**Published:** 2025-10-23

**Authors:** Daniel Marius Duda Seiman, Petru Bucuras, Nilima Rajpal Kundnani, Abhinav Sharma, Maria Rada, Nicolae Albulescu, Victor Buciu, Dana Movila, Dana Emilia Velimirovici, Anca Raluca Dinu

**Affiliations:** 1 Department VI—Cardiology, University Clinic of Internal Medicine and Ambulatory Care, Prevention and Cardiovascular Recovery, “Victor Babes” University of Medicine and Pharmacy, 300041 Timisoara, Romania; daniel.duda-seiman@umft.ro (D.M.D.S.); sharma.abhinav@umft.ro (A.S.); dana.movila@umft.ro (D.M.); dana.velimirovici@umft.ro (D.E.V.); 2 Research Centre of Timisoara Institute of Cardiovascular Diseases, “Victor Babes” University of Medicine and Pharmacy, 300041 Timisoara, Romania; 3 Department of Nephrology, “Pius Brînzeu” Emergency County Hospital, 300723 Timisoara, Romania; 4 Doctoral School, “Victor Babes” University of Medicine and Pharmacy Timisoara, E. Murgu Square, No. 2, 300041 Timisoara, Romania; victor.buciu@umft.ro; 5 Division of Internal Medicine II—Cardiology, “Victor Babes” University of Medicine and Pharmacy, 300041 Timisoara, Romania; nicolae.albulescu@umft.ro; 6 Centre for Molecular Research in Nephrology and Vascular Disease, “Victor Babes” University of Medicine and Pharmacy, 300041 Timisoara, Romania; 7 Department of Cardiology, “Pius Brînzeu” Emergency County Hospital, 300723 Timisoara, Romania; 8 Department XVI, Medical Recovery, “Victor Babes” University of Medicine and Pharmacy, 300041 Timisoara, Romania; dinu.anca@umft.ro; 9 Research Center for Assessment of Human Motion and Functionality and Disability, “Victor Babes” University of Medicine and Pharmacy, Eftimie Murgu Square, No. 2, 300041 Timisoara, Romania; 10 “Pius Brinzeu” Emergency Clinical County Hospital, Bld Liviu Rebreanu, No. 156, 300723 Timisoara, Romania

**Keywords:** chronic kidney disease, educational attainment, social determinants of health, eGFR decline, kidney replacement therapy, cardio-renal-metabolic syndrome

## Abstract

Background: Chronic kidney disease (CKD) progression is shaped not only by biological risk factors but also by social determinants of health. Educational attainment is a key socioeconomic indicator, yet data from Eastern Europe remain limited. Methods: We conducted a retrospective cohort study including 428 adults with cardio-renal-metabolic (CRM) syndrome having CKD stages G3a–G4 enrolled between 2022 and 2024 and followed until December 2024. Patients were stratified by educational attainment using the International Standard Classification of Education (ISCED 2011): low (ISCED 0–2) vs. high (ISCED 3–8). The primary outcome was a composite of a ≥40% decline in estimated glomerular filtration rate (eGFR) or initiation of kidney replacement therapy (KRT). Secondary outcomes were eGFR slope, doubling of urine albumin-to-creatinine ratio (uACR), and KRT initiation. Results: Of 428 patients, 245 (57.2%) had low education. These patients had lower use of renoprotective therapies. During a median follow-up of 32 months, 88 primary outcome events occurred: 66 (27%) in the low education group vs. 22 (12%) in the high education group. In adjusted analyses, low education remained independently associated with the primary outcome (HR 1.47, 95% CI 1.04–2.36, *p* = 0.04). The annual eGFR decline was steeper in patients with low education (−3.0 vs. −2.1 mL/min/1.73 m^2^/year, *p* < 0.001), and doubling of uACR was more frequent (24% vs. 15%, HR 1.47, 95% CI 1.02–2.19, *p* = 0.02). Conclusions: In a Romanian CRM-CKD cohort, lower educational attainment was an independent predictor of faster kidney function decline and adverse renal outcomes. Beyond reflecting individual disadvantage, educational status in Eastern Europe highlights systemic inequities in access to nephrology care and therapies. Incorporating education into risk stratification and implementing equity-focused interventions may improve CKD outcomes in disadvantaged populations.

## 1. Introduction

Chronic kidney disease (CKD) represents a major global health challenge, affecting approximately 10% of the adult population and contributing significantly to cardiovascular morbidity, premature mortality, and healthcare expenditure [1,2]. Despite advances in renoprotective therapies, many patients experience progressive decline in kidney function, ultimately requiring dialysis or transplantation [3]. Traditional risk factors such as diabetes, hypertension, proteinuria, and cardiovascular disease explain part of this variability in outcomes, but increasing evidence suggests that social determinants of health, including socioeconomic status and educational attainment, play a critical role [1,2,4].

As highlighted by most recent guidelines, CKD stage 3a corresponds to eGFR 45–59 mL/min/1.73 m^2^, stage 3b to 30–44, and stage 4 to 15–29. These reflect moderate-to-severe reduction in kidney function [5].

Education, in particular, has been identified as a powerful and consistent predictor of health outcomes across a wide range of chronic diseases. In cardiovascular medicine, lower educational attainment has been associated with increased incidence of coronary artery disease, stroke, and mortality, even after adjustment for traditional risk factors [6]. In oncology, patients with limited education are more likely to present with advanced disease and have lower survival rates. In nephrology, lower education has been linked to delayed diagnosis of CKD, lower awareness of disease status, reduced adherence to medical recommendations, and poorer long-term outcomes, including accelerated progression to kidney failure [7,8,9].

The mechanisms underlying these associations are complex and multifactorial. Lower educational attainment is often associated with limited health literacy, reduced ability to navigate healthcare systems, lower socioeconomic resources, and increased exposure to adverse lifestyle and environmental factors [2,10,11]. These factors may lead to delayed initiation of nephroprotective therapy, suboptimal blood pressure (mmHg) and glycemic control (mg/dL), and reduced uptake of emerging treatments such as sodium–glucose cotransporter-2 (SGLT2) inhibitors. Furthermore, education may influence patient–physician communication, shared decision-making, and adherence to complex treatment regimens [12,13,14,15].

While prior studies from Western Europe and North America have consistently demonstrated that lower education is linked to poorer renal outcomes [16,17,18], data from Eastern Europe remain scarce. This region is marked by pronounced socioeconomic disparities, high out-of-pocket health expenditures, and uneven access to nephrology care. Romania, in particular, combines a high prevalence of CKD with one of the lowest rates of guideline-based therapy uptake in Europe, making it an important setting to investigate the role of educational attainment [19,20].

We therefore aimed to examine whether lower educational attainment is independently associated with faster kidney function decline and adverse renal outcomes in a Romanian CKD cohort.

We hypothesized that patients with lower educational attainment, defined as below high school graduate according to ISCED 2011, would experience faster kidney function decline, measured by estimated glomerular filtration rate (eGFR, mL/min/1.73 m^2^/year), and higher risk of clinically significant renal outcomes, including ≥40% eGFR decline or need for kidney replacement therapy (KRT), independent of comorbidities and treatment factors.

## 2. Methods

This retrospective cohort analysis was conducted and included patients between 2022 and 2024, with follow-up completed until December 2024, ensuring a minimum of 24 months of observation for all participants.

### 2.1. Study Population

Eligible patients were identified through the hospital’s electronic medical records system. Inclusion criteria were: cardio-renal metabolic syndrome, age ≥ 18 years, diagnosis of CKD stage G3a–G4 confirmed by at least two eGFR values (<60 mL/min/1.73 m^2^) [5] measured ≥90 days apart, and a documented education level. Patients were excluded if they were receiving dialysis at baseline, had undergone kidney transplantation, had polycystic kidney disease, or experienced acute kidney injury (AKI) within 30 days of baseline assessment [21].

Enrollment occurred between 1 January 2022 and 31 December 2022, with follow-up until 31 December 2024, ensuring ≥24 months for all participants. 

Cardio-renal-metabolic syndrome was defined as the coexistence of ≥2 of the following: diabetes mellitus, chronic kidney disease stage ≥ 3a, and cardiovascular disease (ischemic heart disease, heart failure, or cerebrovascular disease).

### 2.2. Exposure

Educational attainment was self-reported by patients at hospital admission and recorded in administrative files by trained admission staff. These data were subsequently reviewed and harmonized according to ISCED 2011 classifications [22]. Where information was missing or unclear, it was cross-checked with patient charts or prior admissions. Patients were categorized into two groups: low education (ISCED 0–2: primary or incomplete lower secondary education) and high education (ISCED 3–8: completed high school, vocational, tertiary, or postgraduate education).

### 2.3. Outcomes

The primary outcome was a composite endpoint of sustained ≥40% decline in eGFR (mL/min/1.73 m^2^) from baseline or initiation of KRT (dialysis or kidney transplantation). Secondary outcomes included: annualized eGFR slope (mL/min/1.73 m^2^/year), doubling of urine albumin-to-creatinine ratio (uACR, mg/g) and initiation of KRT (dialysis or transplantation) considered as a separate outcome.

### 2.4. Data Collection and Management

Clinical data were collected retrospectively from electronic health records. Variables included demographics (age in years, sex), comorbidities (diabetes, hypertension, cardiovascular disease), laboratory measures (eGFR in mL/min/1.73 m^2^, uACR in mg/g), systolic blood pressure (mmHg), body mass index (kg/m^2^), smoking status (current, former, never), and medication use (renin–angiotensin–aldosterone system [RAAS] inhibitors, SGLT2 inhibitors, statins).

Data extraction followed a two-stage process: initial abstraction by one investigator and independent verification by another. Discrepancies were adjudicated through consensus review. Laboratory results were validated against hospital laboratory information systems to ensure accuracy. All identifiable patient data were pseudonymized at the time of extraction in compliance with the General Data Protection Regulation (GDPR, EU 2016/679). Study codes replaced personal identifiers, and the re-identification key was stored in a secure, encrypted file accessible only to the principal investigator. Data were maintained on password-protected institutional servers with access restricted to the study team.

Missing data were rare (<3% for all covariates). Patients with missing education data were excluded. For other variables, complete case analysis was used, as the proportion of missingness was low and not clustered by outcome or exposure status. We acknowledge that most of our data stands of KRT follow patients on dialysis, as our regional center does not internally practice kidney transplantation.

### 2.5. Statistical Analysis

Data entry and cleaning were performed using Microsoft Excel 2016 (Microsoft Corp., Redmond, WA, USA). Preliminary descriptive statistics and database management were carried out in SPSS Statistics version 26 (IBM Corp., Armonk, NY, USA). Advanced analyses were performed in R version 4.3.3 (R Foundation for Statistical Computing, Vienna, Austria).

Continuous variables were assessed for normality using the Shapiro–Wilk test and expressed as mean ± standard deviation (SD) or median with interquartile range (IQR), depending on distribution. Between-group comparisons employed independent-samples *t*-tests for normally distributed variables and Mann–Whitney U tests for skewed distributions. Categorical variables were summarized as counts and percentages and compared with chi-squared tests.

Time-to-event analyses for the primary and secondary endpoints were performed using Cox proportional hazards regression. Proportional hazards assumptions were evaluated with Schoenfeld residuals. Covariates were prespecified based on clinical relevance and prior literature linking them to CKD progression (KDIGO guidelines, major cohort studies), rather than data-driven selection: age (years), sex, baseline eGFR (mL/min/1.73 m^2^), baseline uACR (mg/g), diabetes (yes/no), hypertension (yes/no), cardiovascular disease (yes/no), systolic blood pressure (mmHg), body mass index (kg/m^2^), smoking (yes/no), and medication use (RAAS inhibitors, SGLT2 inhibitors, statins). Kaplan–Meier survival curves were generated for the primary composite endpoint. A single multivariable model was prespecified including all covariates. No stepwise or separate models were run; reported HRs represent mutually adjusted effects.

Longitudinal kidney function decline was modeled using linear mixed-effects regression, incorporating random intercepts and slopes to account for within-patient correlation. Sensitivity analyses excluded patients with baseline eGFR < 20 mL/min/1.73 m^2^ to minimize bias from imminent dialysis initiation.

Ethical approval for the study was obtained from the Institutional Review Ethics Committee of Institute of Cardiovascular Diseases, Timisoara (Ref. No. 7319). Given the retrospective design, informed consent was waived, and all procedures complied fully with national legislation and GDPR regulations. The study was conducted in accordance with the Declaration of Helsinki and national legislation.

## 3. Results

### 3.1. Baseline Characteristics

A total of 428 patients with CKD stages G3a–G4 and having cardio-renal-metabolic syndrome were included in the study, of whom 245 (57.2%) had low educational attainment (ISCED 0–2) and 183 (42.8%) had higher education (ISCED 3–8). Table 1 summarizes baseline characteristics. Patients in the low education group were significantly older (mean age 67.1 ± 9.8 years vs. 61.2 ± 10.4 years, *p* < 0.001). Baseline estimated glomerular filtration rate (eGFR, mL/min/1.73 m^2^) was similar between groups (41.3 ± 9.2 vs. 42.8 ± 8.7, *p* = 0.11). Median urine albumin-to-creatinine ratio (uACR, mg/g) was higher in patients with lower education (380 [150–650] vs. 270 [120–540], *p* = 0.03).

Although mean eGFR values were similar, median albuminuria was nearly 40% higher in the low education group, suggesting greater disease burden at baseline.

To account for the age component of eGFR, baseline serum creatinine values were also compared, which did not differ significantly between groups. Values were similar (1.82 ± 0.6 mg/dL vs. 1.79 ± 0.5 mg/dL, *p* = 0.46), confirming that kidney function at baseline was comparable.

Patients with low education were less likely to receive renin–angiotensin–aldosterone system (RAAS) inhibitors (56.7% vs. 68.3%, *p* = 0.01) or sodium–glucose cotransporter-2 (SGLT2) inhibitors (13.1% vs. 24.0%, *p* = 0.004). Mean systolic blood pressure (mmHg) and body mass index (BMI, kg/m^2^) did not differ significantly between groups.

Of the 168 patients with diabetes (39.2% of the cohort), 74 (17.3%) had documented diabetic nephropathy, defined by persistent macroalbuminuria and/or concomitant diabetic retinopathy.

### 3.2. Primary and Secondary Outcomes

During a median follow-up of 32 months (IQR: 28–36), 88 patients experienced the primary composite outcome (≥40% eGFR decline or initiation of kidney replacement therapy). Event rates were significantly higher in the low education group (66/245, 27.0%) compared to the high education group (22/183, 12.0%). The unadjusted hazard ratio (HR) for the low vs. high education group was 1.58 (95% CI 1.02–2.45, *p* = 0.04).

The annualized eGFR slope was significantly steeper among patients with low education (−3.0 ± 0.9 mL/min/1.73 m^2^/year) compared with those with high education (−2.1 ± 0.8 mL/min/1.73 m^2^/year, *p* < 0.001). Doubling of uACR (mg/g) occurred in 24.1% of the low education group versus 15.3% of the high education group (HR 1.47, 95% CI 1.02–2.19, *p* = 0.02). Initiation of kidney replacement therapy (dialysis or transplantation) was more frequent in the low education group (11.0% vs. 7.1%), although the difference did not reach statistical significance (HR 1.38, 95% CI 0.70–2.73, *p* = 0.18). Those results are further illustrated in Table 2.

The absolute difference in primary outcome incidence was 15% between groups (27% vs. 12%), translating into one additional adverse event for every ~7 patients with lower education compared with higher education.

### 3.3. Multivariable Cox Regression

When adjusting for age (years), sex, baseline eGFR (mL/min/1.73 m^2^), baseline uACR (mg/g), diabetes (yes/no), hypertension (yes/no), cardiovascular disease (yes/no), systolic blood pressure (mmHg), BMI (kg/m^2^), smoking status (yes/no), and use of RAAS inhibitors, SGLT2 inhibitors, and statins, low educational attainment remained independently associated with the composite primary outcome (adjusted HR 1.47, 95% CI 1.04–2.36, *p* = 0.04) (Table 3).

In addition to education, diabetes mellitus was independently associated with the primary composite outcome (HR 1.39, 95% CI 1.01–1.91, *p* = 0.045), underscoring its well-established role as a driver of CKD progression.

### 3.4. Survival Analysis and Kidney Function Trajectories

**Figure 1:**
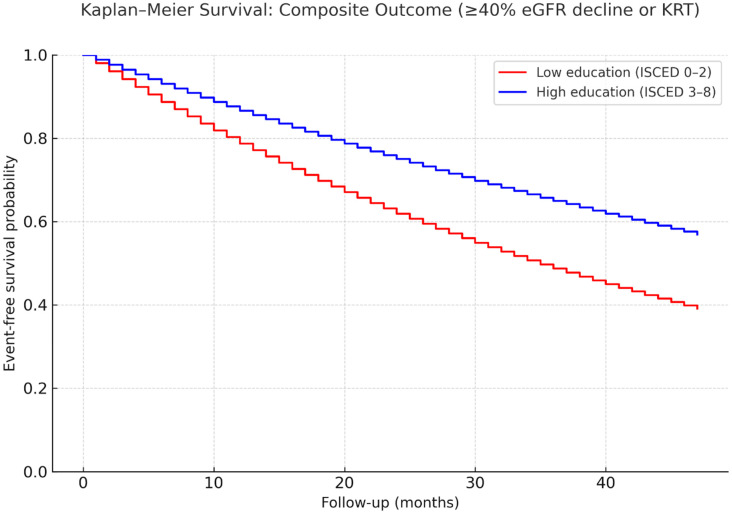
Kaplan–Meier survival curves for the composite outcome (≥40% eGFR decline or initiation of kidney replacement therapy) stratified by educational attainment. Survival probabilities were estimated using the Kaplan–Meier method and compared between groups with the log-rank test. Hazard ratios were derived from Cox proportional hazards models adjusted for baseline covariates (age, sex, baseline eGFR, uACR, diabetes, RAAS and SGLT2 inhibitor use). eGFR: estimated glomerular filtration rate; KRT: kidney replacement therapy.

Kaplan–Meier survival analysis demonstrated that patients with low educational attainment had a significantly higher cumulative incidence of the composite outcome (≥40% eGFR decline or initiation of kidney replacement therapy) compared with those with higher education. The divergence between groups became evident after the first 12 months of follow-up and widened progressively during the observation period (Figure 1).

**Figure 2:**
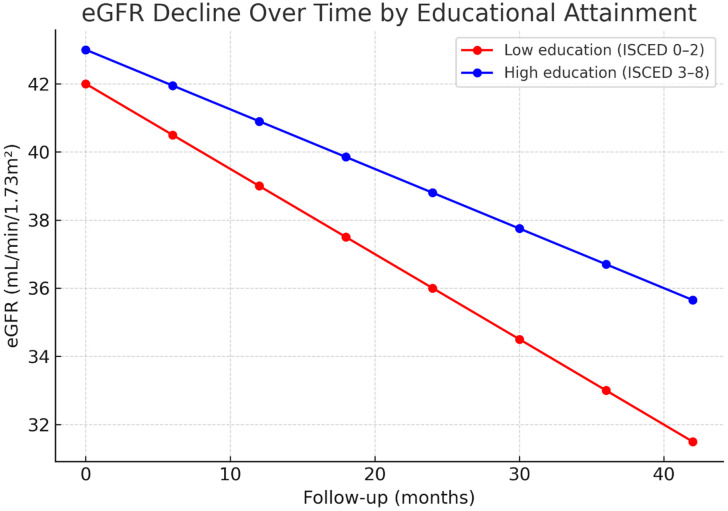
Estimated decline in eGFR over time by educational attainment. Longitudinal kidney function trajectories were modelled using linear mixed-effects regression with random intercepts and slopes. Mean estimated eGFR values (mL/min/1.73 m^2^) at 6-month intervals are plotted with 95% confidence intervals. Patients in the low education group exhibited a significantly steeper decline (−3.0 vs. −2.1 mL/min/1.73 m^2^/year; between-group difference *p* < 0.001). eGFR: estimated glomerular filtration rate.

To further illustrate differences in kidney function trajectories, repeated measures of eGFR (mL/min/1.73 m^2^) were modelled using linear mixed-effects regression. Patients in the low education group exhibited a steeper decline in eGFR compared with those in the high education group, with slopes of −3.0 versus −2.1 mL/min/1.73 m^2^/year, respectively. The trajectories of mean eGFR decline across 48 months of follow-up are displayed in Figure 2.

## 4. Discussion

In this retrospective cohort study of patients with CRM syndrome having CKD stages G3a–G4 treated at a tertiary referral center in Romania, we found that lower educational attainment was associated with a significantly higher risk of clinically relevant kidney outcomes, including ≥40% decline in eGFR (mL/min/1.73 m^2^) or initiation of kidney replacement therapy (KRT). Patients with lower education also had a steeper annualized eGFR decline and higher likelihood of albuminuria progression. These associations persisted even after adjustment for baseline kidney function, comorbidities, and pharmacologic therapy, underscoring the independent contribution of educational attainment to CKD progression.

To our knowledge, this is the first study from Eastern Europe to demonstrate the independent impact of educational attainment on CKD progression, providing important evidence from a region characterized by substantial healthcare and socioeconomic inequalities. We emphasize that our findings highlight risk, but not direct causality, because of this study’s retrospective nature. This will further on be discussed on limitations. Our findings align closely with previous studies conducted in Western populations. In Chronic Renal Insufficiency Cohort Study (CRIC), education was collected, but progression was mainly predicted by albuminuria [23]. Baseline reports noted associations of lower eGFR with socioeconomic disadvantage, including lower income and education, but did not directly test education as an independent predictor [24]. Thus, our study adds specific evidence on education as an independent risk factor. Similarly, a registry-based study across several European countries demonstrated that patients with lower education were more likely to experience faster CKD progression and less likely to receive guideline-recommended therapies [24,25]. Morton et al. reported that lower education was associated not only with poorer renal outcomes but also with reduced quality of life in moderate-to-severe CKD [26].

The magnitude of effect observed in our cohort (adjusted HR 1.47) is consistent with these international findings [6,16,24], suggesting that educational disparities exert a robust influence on CKD trajectories across diverse settings. Importantly, the Romanian context, with lower uptake of nephroprotective therapies and wider socioeconomic inequalities, provides a distinct lens through which these associations can be understood [20].

The impact of education on health outcomes is not unique to kidney disease. In cardiovascular medicine, lower educational attainment has been consistently linked to higher incidence of coronary heart disease, stroke, and cardiovascular mortality [27,28,29,30]. In oncology, patients with lower education often present with more advanced disease and have worse survival, likely reflecting a combination of delayed diagnosis, reduced screening uptake, and disparities in treatment access [31,32,33,34,35]. Even in infectious diseases, such as HIV, education has been shown to predict adherence to antiretroviral therapy and long-term virological suppression [36,37,38,39].

Although BMI did not differ significantly between groups, low literacy and education are often associated with obesity and poor nutrition, which may mediate CKD progression, as shown in prior cohorts [40].

Taken together, these findings underscore that education serves as a powerful upstream determinant of health, shaping individual behaviours, healthcare utilization, and access to novel therapies [41,42]. Our results extend this evidence into nephrology, highlighting that lower educational attainment is not only a marker of disadvantage but an actionable risk factor that could inform targeted interventions.

### 4.1. Mechanisms and Interpretation

Several interconnected mechanisms may explain the observed association between lower educational attainment and accelerated CKD progression in our cohort. Health literacy is a central factor: patients with limited education often have greater difficulty understanding their disease, the importance of regular follow-up, and the rationale for complex multidrug regimens, which can reduce adherence and impair patient–physician communication. In recent years, with the development of better and more complex therapies, the once straightforward treatment has now become a complex, interdisciplinary road [43,44,45,46]. In our study, patients with lower education were also less likely to receive renin–angiotensin–aldosterone system (RAAS) inhibitors and sodium–glucose cotransporter-2 (SGLT2) inhibitors, therapies strongly recommended by KDIGO guidelines, suggesting structural or behavioural barriers to the uptake of reno protective treatment. In our cohort, prescription of RAAS and SGLT2 inhibitors was significantly lower in patients with lower education, reflecting early disparities in access to nephroprotective therapy.

Beyond health literacy and pharmacotherapy, psychosocial stress is disproportionately prevalent in disadvantaged populations and may contribute biologically to kidney injury [47,48,49,50]. Chronic activation of the hypothalamic–pituitary–adrenal axis and sympathetic nervous system can promote glomerular hypertension, systemic inflammation, and endothelial dysfunction, all of which accelerate nephron loss [51,52,53]. Nutritional and environmental exposures provide an additional pathway: individuals with lower education are more likely to consume high-sodium, processed diets and to experience greater exposure to occupational or environmental toxins, both of which worsen albuminuria and accelerate CKD progression [54,55,56,57].

The independent contribution of diabetes observed in our multivariable model is consistent with its established role as the leading cause of CKD progression globally [5].

Taken together, these mechanisms indicate that educational attainment is not simply a socioeconomic marker but an upstream determinant with direct biological and clinical consequences for CKD outcomes.

### 4.2. Particularities of the Low Resource Settings

These mechanisms may be particularly pronounced in Eastern Europe, where structural inequalities in healthcare delivery amplify the effects of education on disease outcomes. Romania exemplifies this context, with one of the highest prevalences of CKD in Europe and persistent gaps in access to nephrology services [58,59,60]. High out-of-pocket healthcare expenditures, uneven distribution of specialist care, and rural–urban disparities create substantial barriers to timely diagnosis and treatment. Educational inequalities remain profound, with nearly one-third of adults completing only primary or lower secondary education, and this disadvantage is often compounded by residence in underserved rural areas [58].

In such settings, low educational attainment does not merely reflect an individual’s social position but serves as a proxy for broader systemic disadvantage. Limited health system capacity, combined with socioeconomic barriers, likely exacerbates the treatment gaps observed in our study, such as lower use of RAAS and SGLT2 inhibitors. These contextual factors help explain why educational disparities in Romania may exert a stronger influence on CKD progression compared with higher-income Western countries, underscoring the importance of tailoring both clinical practice and health policy to the regional context.

### 4.3. Clinical and Public Health Implications

Our findings carry several implications for both clinical practice and health policy. From a clinical perspective, educational attainment should be considered a risk stratification factor, similar in magnitude to diabetes or cardiovascular disease. Patients with lower education may benefit from closer monitoring of kidney function and albuminuria, as well as simplified and reinforced counselling strategies to improve adherence. Tailored communication approaches, including the use of visual aids and structured educational tools, could help bridge health literacy gaps in this population [61,62]. We emphasise the need for personalised teaching programmes in order to better the understanding and therapeutic adherence in lower education populations. This has been priorly studied and proven useful in western cohorts, either through specific educational strategies, online courses or both [10,63,64].

At the health system level, addressing inequities in access to evidence-based therapies is critical. The lower use of RAAS and SGLT2 inhibitors observed in our study highlights the need for policies that ensure affordability and integration of these medications into national reimbursement schemes. In Romania, prior use of SGLT2 inhibitors has been available since 2012, but only since 2023 has it been covered by national health insurance [20,65,66], despite its efficiency having been proven for almost a decade [67,68]. Targeted outreach to rural and underserved areas, where lower educational attainment is most prevalent, may improve timely diagnosis and referral.

From a broader public health perspective, integrating educational status into CKD registries, screening programs, and risk prediction models could enhance the early identification of vulnerable patients. Public health campaigns and community-based initiatives should be culturally adapted and designed to address both literacy and resource barriers. Public health campaigns and community-based initiatives must be culturally adapted and literacy-sensitive to effectively reach underserved populations. Evidence shows that theory-driven, person-centered, and culturally tailored interventions yield stronger engagement among disadvantaged groups. Building health literacy capacity at the system level ensures sustainability [69,70,71]. Recognizing educational attainment as a determinant of CKD outcomes offers an opportunity to design interventions that are not only medically effective but also equity-oriented.

### 4.4. Limitations

This study has several limitations that should be acknowledged. First, educational attainment was extracted from electronic health records, where it may have been self-reported or incompletely documented, raising the possibility of misclassification bias. Second, although education is a robust socioeconomic indicator, it does not fully capture other dimensions such as household income, occupational exposures, or wealth, which may independently influence CKD outcomes. Third, medication use was assessed based on electronic prescription data rather than long term verified adherence, which could underestimate disparities in real-world treatment uptake. Fourth, unmeasured lifestyle factors such as diet quality, physical activity, and use of traditional remedies could not be accounted for, though these may differ substantially by educational background. Fifth, although age is embedded in eGFR estimation, our sensitivity analysis using serum creatinine confirmed group comparability. Finally, the single-center design and restriction to a Romanian cohort limit generalizability to other populations with different health systems or socioeconomic contexts. Causality cannot be inferred due to the observational design. Despite these limitations, our findings are strengthened by detailed clinical characterization, longitudinal follow-up of at least 24 months, and consistency with international literature.

### 4.5. Future Perspectives

Despite these limitations, our study has several implications for research and practice. Future studies should integrate multiple socioeconomic indicators, including income, occupation, and rurality, into CKD risk prediction models. Interventional studies are also needed to determine whether tailored education programs, community outreach, or policy-level initiatives can reduce disparities in CKD outcomes. The growing role of digital health may offer new opportunities for patient education, but access to technology itself may mirror educational disparities, requiring careful implementation.

At a policy level, our findings highlight the need for health systems in Eastern Europe to integrate social determinants of health into CKD care pathways. Programs that ensure equitable access to RAAS inhibitors and SGLT2 inhibitors, regardless of socioeconomic background, may be particularly impactful. From a clinical perspective, nephrologists should recognize educational attainment as an important risk factor, similar in magnitude to diabetes or cardiovascular disease, and prioritize more intensive monitoring and counselling in these patients.

## 5. Conclusions

In this Romanian cohort of patients with CKD stages G3a–G4, lower educational attainment was independently associated with faster decline in kidney function, greater progression of albuminuria, and a higher risk of clinically relevant renal outcomes. These associations persisted after adjustment for comorbidities and therapy use, underscoring education as a powerful determinant of CKD progression. Beyond reflecting individual disadvantage, educational status in Eastern Europe also signals systemic barriers to care, including limited access to nephroprotective therapies and specialist services.

Clinically, educational background should be incorporated into risk stratification and guide more intensive monitoring and tailored counselling. At the health system level, improving equitable access to RAAS and SGLT2 inhibitors and strengthening outreach to underserved populations may reduce disparities.

Future studies should evaluate whether targeted interventions, such as simplified patient education tools, community outreach, and policy-driven medication subsidies, can mitigate these disparities. Addressing the social and biological consequences of lower educational attainment represents not only a clinical necessity but also an opportunity to advance equity in kidney care across diverse populations.

## Figures and Tables

**Table 1:** Baseline characteristics of study participants by educational attainment.

Characteristic	Low Education (n = 245)	High Education (n = 183)	*p*-Value
Age (years)	67.1 ± 9.8	61.2 ± 10.4	<0.001
Male sex (n, %)	129 (52.7)	91 (49.7)	0.54
Baseline eGFR (mL/min/1.73 m^2^)	41.3 ± 9.2	42.8 ± 8.7	0.11
uACR (mg/g), median [IQR]	380 [150–650]	270 [120–540]	0.03
Hypertension (n, %)	205 (83.7)	142 (77.6)	0.12
Systolic blood pressure (mmHg)	136 ± 17	134 ± 16	0.21
Diabetes Mellitus (n,%)	108 (44.1)	60 (32.8)	0.02
BMI (kg/m^2^)	28.5 ± 4.7	27.9 ± 4.4	0.24
Current smoking (n, %)	52 (21.2)	33 (18.0)	0.41
RAAS inhibitor use (n, %)	139 (56.7)	125 (68.3)	0.01
SGLT2 inhibitor use (n, %)	32 (13.1)	44 (24.0)	0.004
Statin use (n, %)	106 (43.3)	91 (49.7)	0.19

Footnote: Continuous variables expressed as mean ± standard deviation or median [interquartile range]; compared with Student’s *t*-test or Mann–Whitney U test, as appropriate. Categorical variables compared with chi-squared test. eGFR: estimated glomerular filtration rate; uACR: urine albumin-to-creatinine ratio; BMI: body mass index; RAAS: renin–angiotensin–aldosterone system; SGLT2: sodium–glucose cotransporter-2.

**Table 2:** Outcome events by education group.

Outcome	Low Education (n = 245)	High Education (n = 183)	HR (95% CI)	*p*-Value
Primary outcome (≥40% eGFR decline or KRT)	66 (27.0%)	22 (12.0%)	1.58 (1.02–2.45)	0.04
eGFR slope (mL/min/1.73 m^2^/year)	−3.0 ± 0.9	−2.1 ± 0.8		<0.001
Doubling of uACR (mg/g)	59 (24.1%)	28 (15.3%)	1.47 (1.02–2.19)	0.02
KRT initiation (n, %)	27 (11.0%)	13 (7.1%)	1.38 (0.70–2.73)	0.18

Footnote: Hazard ratios estimated with Cox proportional hazards regression. eGFR slope derived from linear mixed-effects models. eGFR: estimated glomerular filtration rate; KRT: kidney replacement therapy; uACR: urine albumin-to-creatinine ratio.

**Table 3:** Multivariable Cox regression for the primary composite outcome.

Variable	Adjusted HR (95% CI)	*p*-Value
Low vs. high education	1.47 (1.04–2.36)	0.04
Age (per year)	1.02 (1.00–1.04)	0.05
Male sex	1.09 (0.72–1.64)	0.68
Baseline eGFR (per 5 mL/min/1.73 m^2^)	0.87 (0.78–0.96)	0.007
Log uACR (per doubling, mg/g)	1.21 (1.08–1.35)	0.002
Hypertension	1.12 (0.63–2.00)	0.71
Diabetes Mellitus	1.39 (1.01–1.91)	0.045
RAAS inhibitor use	0.74 (0.50–1.10)	0.14
SGLT2 inhibitor use	0.68 (0.39–1.20)	0.18

Footnote: Cox regression adjusted for prespecified baseline covariates. HR: hazard ratio; CI: confidence interval; eGFR: estimated glomerular filtration rate; uACR: urine albumin-to-creatinine ratio; RAAS: renin–angiotensin–aldosterone system; SGLT2: sodium–glucose cotransporter-2.

## Data Availability

The original contributions presented in this study are included in the article. Further inquiries can be directed to the corresponding author(s).

## Abbreviations

AKI	Acute kidney injury
BMI	Body mass index
CI	Confidence interval
CKD	Chronic kidney disease
CRIC	Chronic Renal Insufficiency Cohort
CRM	cardio-renal-metabolic syndrome
ESKD	End-stage kidney disease
eGFR	Estimated glomerular filtration rate
GDPR	General Data Protection Regulation
HR	Hazard ratio
ISCED	International Standard Classification of Education
IQR	Interquartile range
KRT	Kidney replacement therapy
KDIGO	Kidney Disease: Improving Global Outcomes
RAAS	Renin–angiotensin–aldosterone system
SD	Standard deviation
SGLT2	Sodium–glucose cotransporter-2
uACR	Urine albumin-to-creatinine ratio
